# Lower Limb Motion Classification of Actions in Confined Environments Based on Multi-Source Signal Fusion and Muscle Symmetry Features

**DOI:** 10.3390/s26165314

**Published:** 2026-08-21

**Authors:** Dingzhe Li, Xiaorong Guan, Zheng Wang, Changlong Jiang, Long He, Xiwang Mao, Qiang Zhou

**Affiliations:** 1 School of Mechanical Engineering, Nanjing University of Science and Technology, No. 200, Xiaolingwei, Nanjing 210094, China; lidingzhe@njust.edu.cn (D.L.); wangzheng19@njust.edu.cn (Z.W.); jiangchanglong@njust.edu.cn (C.J.); helong@zy-cs.com.cn (L.H.); maoxiwang@njust.edu.cn (X.M.); 2 Hangzhou Zhiyuan Research Institute Co., Ltd., No. 188 Yunzhan Road, Hangzhou 310000, China; 3 Air Force Hospital of Eastern Theater Command, Nanjing 210002, China; zq20052006@163.com

**Keywords:** motion classification, multi-source fusion, mutual information, muscle symmetry features, assisted exoskeletons

## Abstract

In recent years, advances in exoskeleton technology have increased the demand for human motion classification in terms of both movement diversity and recognition accuracy, making the effective classification of more complex asymmetric movements increasingly important. This study integrated surface electromyography (sEMG) and inertial measurement unit (IMU) signals and employed mutual information (MI) and muscle symmetry features (MSF) to analyze the characteristic differences between symmetrical muscles in both legs during asymmetric movements, with the aim of improving the accuracy of asymmetric motion classification. sEMG and IMU signals were collected from six movements, including three asymmetric postures: asymmetrical stance, single-knee kneeled position, and crouching advance. The acquired signals were processed through energy envelope analysis, active segment extraction, empirical mode decomposition (EMD), feature extraction, MI extraction, and MSF extraction. The relevance based on weight feature selection (RWFS) combined with conditional mutual information (CMI) method was then applied to reduce feature dimensionality, prioritize features with significant fluctuations, and preserve key characteristics. Finally, the CNN-LSTM-Attention algorithm was used for classification. Experimental results showed that fusing sEMG and IMU signals achieved 96.30% accuracy in lower limb motion recognition. The proposed method improves asymmetric movement classification and may provide a potential basis for exoskeleton motion classification in special environments.

## 1. Introduction

In recent years, exoskeleton technology has gradually been adopted by advanced military forces worldwide. In the military domain, exoskeletons can assist soldiers with lifting and unloading [1], aid them in walking and running on the battlefield [2], and enhance their load-bearing capacity during military missions [3]. However, we hope that exoskeletons can assist soldiers in performing more complex professional actions. Controlling exoskeletons to perform the same actions as soldiers requires precise recognition of human movement. Therefore, through the monitoring, judgment, and identification of the current state via sensors, it is possible to predict the next movement [4,5,6].

There are many sensors currently used for human lower limb motion classification, among which surface electromyography (sEMG) sensors and inertial measurement unit (IMU) sensors are commonly used for identifying motion intentions. Huang H et al. [7] explored the use of sEMG signals combined with pattern recognition to identify the wearer’s movement patterns. They selected actions such as flat ground walking, stepping over obstacles, going up and down stairs, turning left, turning right, and standing still. The results showed that the average classification errors for the four determined phases were 12.4%, 6.0%, 7.5%, and 5.2%, respectively. A study at the University of Michigan used data from five IMUs installed on the subjects’ bodies combined with Convolutional Neural Networks (CNN) to identify gait in walking individuals, achieving an accuracy rate of up to 97% [8]. Guangyi Shi [9] et al. used motion feature vectors collected by IMU sensors for human motion recognition. The selected actions included falling, walking, standing, running, and climbing stairs. Support vector machine (SVM) machine learning methods were used for multi-classification of human motions. The experimental results showed that for the given five different types of movements, the total correct recognition rate was 92%, with a 100% recognition rate for fall detection. Deng Ping [10] et al. attached inertial sensors to the waist of test subjects, collecting acceleration data during seven common gaits, resulting in eight feature values for human posture discrimination. This study only used the waist acceleration of the test subject to identify movements. Due to the single data source, it could only recognize continuous gait movements but was unable to effectively identify typical lower limb joint movements.

Wei C et al. [11] proposed a novel lower limb motion recognition method based on empirical mode decomposition and k-nearest neighbors (KNN) Entropy estimation. They selected four actions: flat ground walking, jumping, and going up and down stairs. Three machine learning classifiers—SVM, KNN, and Bagging—were used to classify the four types of lower limb movements. The experimental results showed that the combination of the SVM classifier and diffusion map (DM) method achieved excellent recognition performance, with an accuracy rate as high as 99.63%. However, the number of selected actions was limited.

As the demand for a greater variety of actions and higher degrees of freedom in lower limb exoskeletons increases, models trained with offline sEMG or IMU signal features have shown high accuracy in specific environments. However, their performance in unfamiliar environments is often affected by unforeseen variables [12]. Single signal sources struggle to meet the precision requirements for motion classification. Multi-modal signal fusion not only helps improve the accuracy of motion classification but also enhances the adaptability and flexibility of exoskeleton systems, thereby better meeting the needs of wearers and improving the functionality of exoskeletons. To develop more efficient and ergonomically designed exoskeleton systems, researchers are exploring multi-source signal fusion techniques as control sources for lower limb exoskeletons to achieve more precise control.

Professor Kazuo Kiguchi and his team [13] demonstrated that combining electroencephalogram (EEG) and electromyography (EMG) signals can more accurately estimate upper limb motion intentions, with an accuracy improvement of 5% compared to using sEMG signals alone. Aly H et al. [14] integrated EEG signals with sEMG signals to study the efficiency of deep learning in signal fusion hybrid systems. The team tested the system on a dataset combining multi-channel EEG signals with multi-channel sEMG signals to decode hand and wrist movements. The results showed that models using fused signals had higher recognition accuracy compared to those using individual signals. However, EEG signals are inconvenient to wear and have significant individual differences, whereas IMU sensors are easy to wear and less susceptible to external environmental noise. Vásconez J et al. [15] proposed a Deep Q-Network (DQN) algorithm based on sEMG and IMU information, capable of effectively recognizing 11 gestures (including static and dynamic gestures). The algorithm performs well in complex environments, with classification and recognition rates of 97.50% and 88.15% for static gestures, respectively. Additionally, Ananda Sankar Kundu et al. [16] developed a gesture recognition system based on sEMG and IMU sensors to control an omnidirectional wheelchair. The system achieved a training accuracy of 94% and a practical application accuracy of 90.5% for dynamic gesture recognition. These studies demonstrate that using sEMG, IMU signals, and deep learning technologies can significantly enhance motion recognition accuracy. Currently, most research on multi-source signal fusion methods focuses primarily on gesture classification, with relatively fewer studies dedicated to lower limb motion classification.

The human lower limb mainly includes the knee joint, hip joint, and ankle joint, which are quite flexible and allow the lower limb to perform many different actions. Precise control of lower limb exoskeletons requires accurate classification of these actions. Human lower limb movements mainly include flat ground walking, going up and down stairs, ascending and descending slopes, squatting and rising, rotational movements, dynamic turns, and backward movements. However, to use these in special scenarios such as low and narrow environments, it is necessary to classify some special lower limb actions, such as crouching advance, asymmetric standing, and single-knee kneeled position.

Asymmetric lower limb movements involve different functional roles and muscle activation patterns between the two legs. Estévez et al. used sEMG and IMU signals to recognize movements including left and right turning, but bilateral asymmetry was not explicitly characterized [17]. Jeong and Chung found that half-kneeling produced a distinct lower limb muscle activation pattern compared with standing and bilateral kneeling [18]. However, the use of bilateral sEMG and IMU information to explicitly characterize and recognize asymmetric actions such as single-knee kneeling remains insufficiently investigated.

Obtaining more inter-muscle feature information allows for more effective discrimination of complex movements. Mutual information (MI) features are now widely applied in biomedical data analysis [19]. MI is an information-theoretic metric capable of effectively capturing both linear and nonlinear dependencies between features and target variables. It is independent of any specific learning model and exhibits excellent generalizability and robustness. Research by Baofeng Guo et al. demonstrates that MI-based sequence selection methods can provide effective solutions for high-dimensional human gait data [20]. Zhaojie Ju et al. proposed a correlation analysis method based on EMG signals to recognize human hand movements [21]. MI measures were used to analyze the sequential patterns recorded by sEMG. The MI values were extracted from the EMG signals. Experimental results showed that incorporating MI features outperformed single features and other multi-feature approaches. MI provided important supplementary information for hand movements, thereby improving recognition performance.

Using multiple source signals to accurately classify movements involves processing a large amount of data. Machine learning methods can effectively handle large datasets. Currently, the main deep learning algorithms used for motion classification include artificial neural network (ANN) [22], recurrent neural network (RNN) [23], k-nearest neighbors (KNN) [24], linear discriminant analysis (LDA), decision tree (TREE) [25], and convolutional neural network–long short-term memory (CNN-LSTM) [26]. These models have limitations when processing sequence data with spatiotemporal correlations. The convolutional neural network–long short-term memory network with an attention (CNN-LSTM-Attention) algorithm can effectively capture spatial and temporal features in the data. It has shown good results in fields such as finance, environment, and fault detection [27,28,29,30,31,32]. In this study, we used the CNN-LSTM-Attention algorithm to classify lower limb movements.

Jinwei Liu et al. [33] proposed a dynamic gesture recognition network based on CNN-LSTM-Attention. First, it extracts spatial features of dynamic gestures at each moment from the gesture sequence. Then, it transforms these features into spatiotemporal features using a recurrent neural network with attention mechanisms. Finally, the transformed features are input into a fully connected neural network for gesture recognition. The dynamic gesture recognition network achieved a recognition rate of 93.5% on the Sahand dataset.

Crouching advance and single-knee kneeled position are frequently used in confined, low-height environments. Asymmetric standing is the standard leg posture for standing shooting, providing better balance, adjusting the center of gravity, and preparing for movement. These actions are commonly seen in disaster sites such as earthquakes or in military operations. Additionally, these three actions are frequently used in maintenance, construction, and agricultural activities. To make exoskeletons more flexible and capable of recognizing human movements in confined, low-height, and other special environments, this study collected sEMG and IMU signals for asymmetric standing, crouching advance, and single-knee kneeled position. Simultaneously, signals for flat ground walking, running, and squatting were collected. To account for differences in behavior between the left and right legs, the study combined sEMG and IMU signals from both legs and used the CNN-LSTM-Attention deep learning algorithm for classification.

The main contributions of this study are as follows. First, a multi-source lower limb motion dataset combining bilateral sEMG and IMU signals was established for six movements, including asymmetric standing, single-knee kneeling, and crouching advance, which are representative actions in confined and low-height environments. Second, muscle symmetry features (MSFs) based on bilateral differences in RMS and MPF were introduced to explicitly characterize the imbalance in muscle activation intensity and frequency distribution between the two legs, while mutual information features were used to describe inter-muscle coordination. Third, the RWFS + CMI feature-selection method was combined with a CNN-LSTM-Attention model to reduce feature redundancy and capture spatial–temporal dependencies, thereby improving the classification of both conventional and asymmetric lower limb movements.

The organization of this paper is as follows: Section 2 provides a detailed introduction to the experimental methods and algorithm selection, including experimental design, data collection, data processing, feature extraction, MI feature extraction and dataset construction. Section 3 introduces the principles of the CNN-LSTM-Attention deep learning algorithm and the data analysis methods. Section 4 compares experimental results under different conditions, followed by the discussion in Section 5 and the conclusions in Section 6.

## 2. Materials and Methods

**Figure 1:**
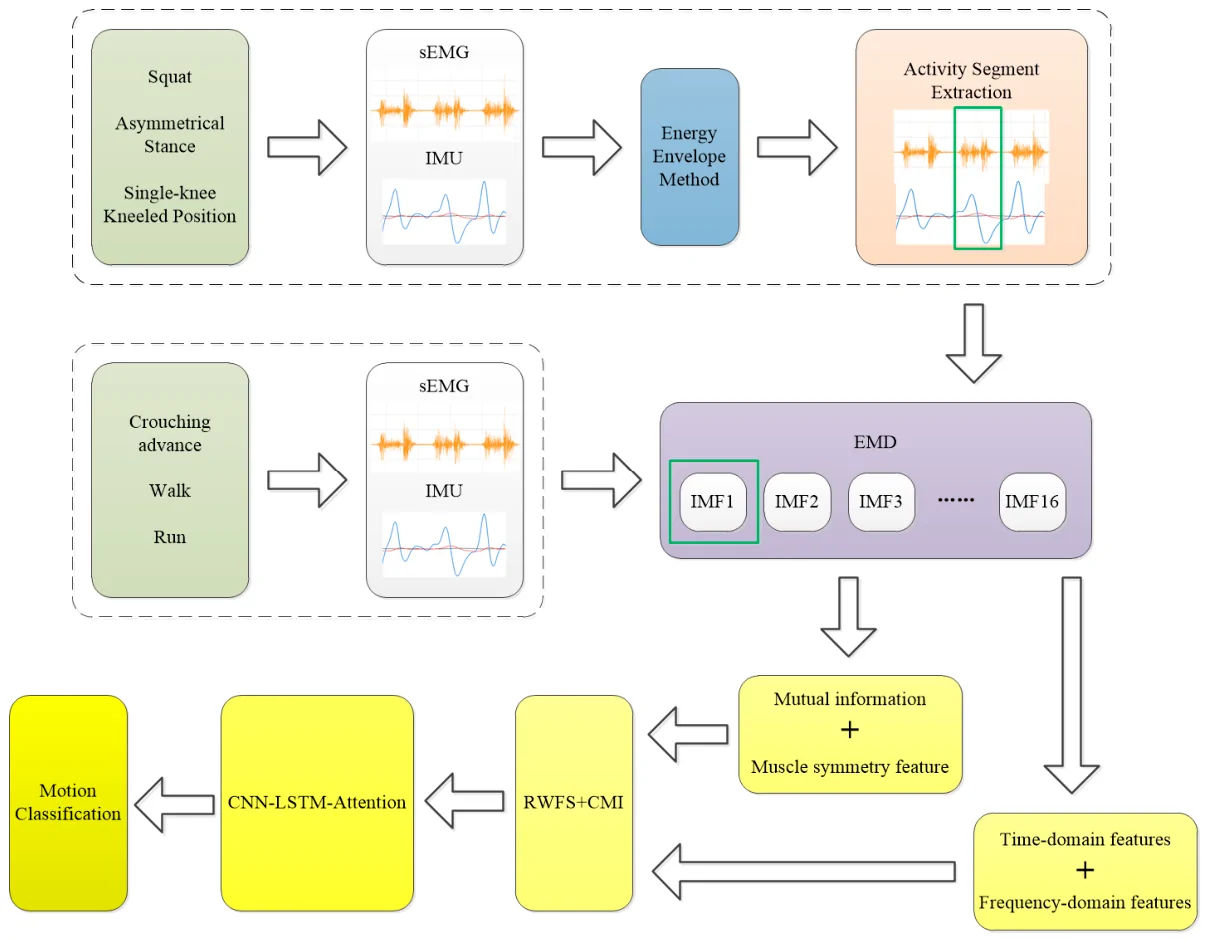
Lower Limb Motion Classification Method Based on sEMG and IMU.

In this section, a motion classification method based on the fusion of sEMG signals and IMU signals was adopted. This method consists of six stages: data acquisition, data preprocessing, activity segment extraction, empirical mode decomposition (EMD), feature extraction, MI feature extraction, relevance based on weight feature selection (RWFS) combined with conditional mutual information (CMI) feature dimensionality reduction and motion classification, and the combination of the fused signal with the CNN-LSTM-Attention algorithm. The specific process is shown in Figure 1. The sEMG signals and IMU signals are fused at the feature level, and then combined with the CNN-LSTM-Attention algorithm. The subsequent sections will provide a detailed explanation of each stage.

### 2.1. Experimental Data Acquisition Scheme

This experiment was approved and consented to by the Ethics Committee of the Nanjing Qixia District Hospital research organization, and informed consent was obtained from all subjects before the experiment. All methods comply with the relevant provisions of the Civil Code of the People’s Republic of China on human experimentation.

In this experiment, three types of lower limb movements in low and narrow special environments were selected: crouching advance, standing to asymmetrical stance, and standing to single-knee kneeled position. Additionally, the sEMG and IMU signals for three types of movements, including flat ground walking, running, and squatting, were collected.

**Figure 2:**
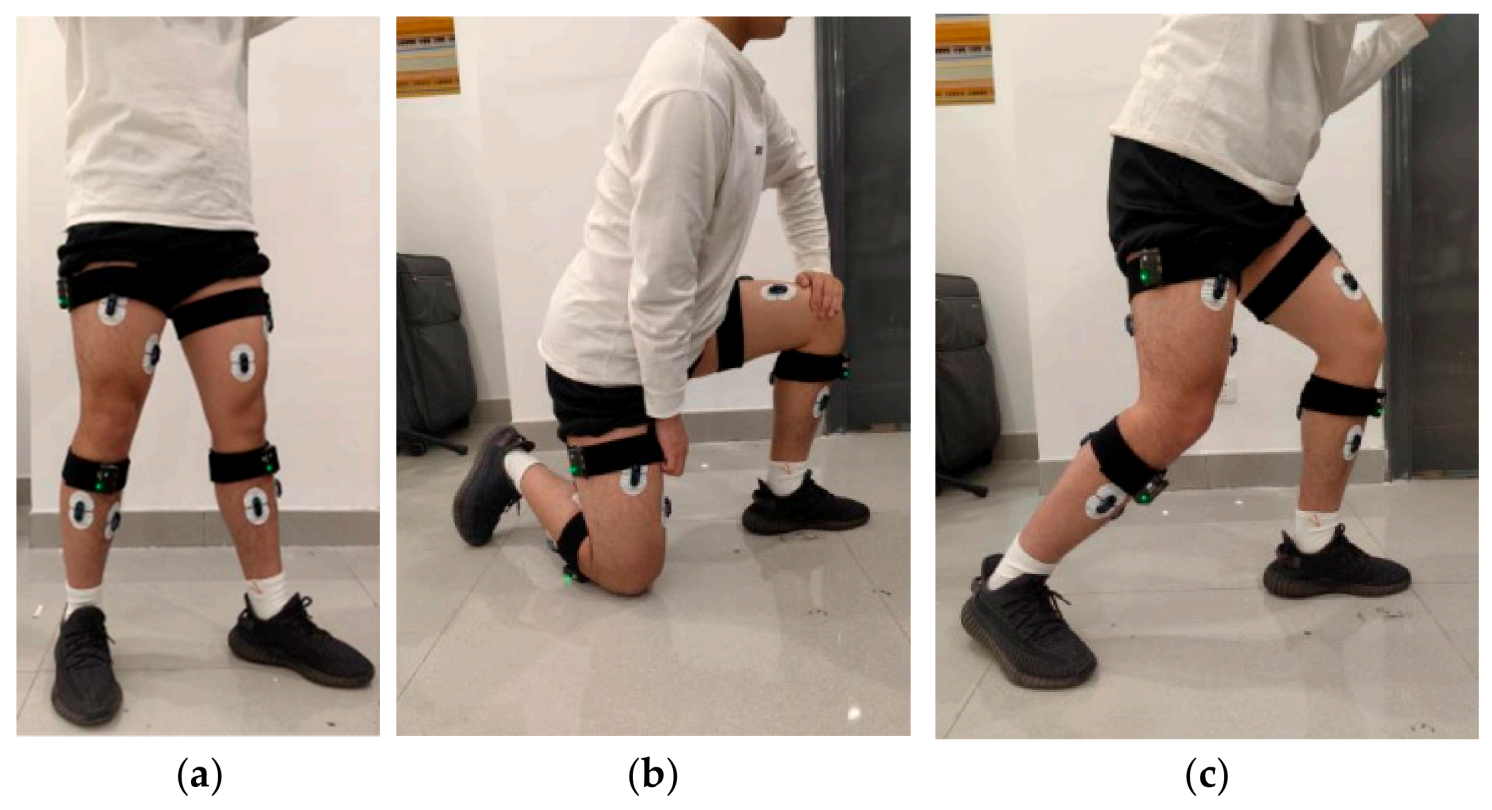
(**a**) asymmetrical stance (**b**) single-knee kneeled position (**c**) Crouching advance.

The characteristics of the asymmetrical stance movement are as follows: the body leans to the left side towards the target and the left foot steps forward about shoulder-width apart, forming a T-shape with the right foot. This posture has good body control and stability and is commonly used by soldiers when shooting while standing. The features of the single-knee kneeled movement are as follows: the body turns slightly to the right, the right knee touches the ground, and the buttocks sit on the right heel. The feet are spread approximately 90 degrees, and the upper body leans slightly forward. The left elbow is supported on the left knee. This posture is often used in handling narrow spaces. The characteristics of the crouching advance movement are as follows: the upper body is tilted forward, both legs are bent, and the movement is made slowly. This posture is commonly used for moving in low and narrow environments. As shown in Figure 2.

**Figure 3:**
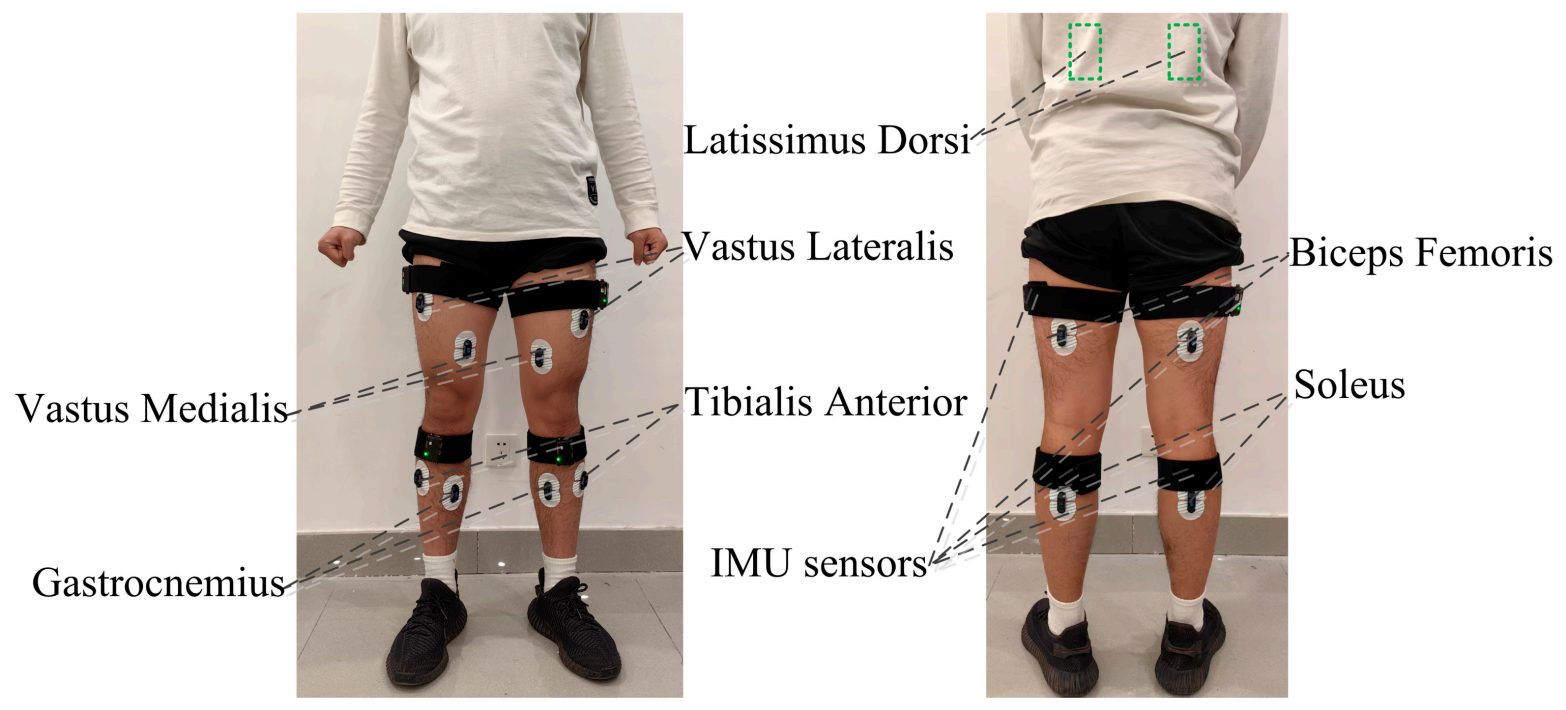
Placement Positions of sEMG and IMU Sensors.

This experiment utilized the Cometa wireless system (Wireless sEMG acquisition device manufactured by Comete, Bareggio, Italy) to collect sEMG signals. This device has a sensing delay of less than 500 microseconds and is equipped with 16 sensors. The sampling rate during the experiment was set to 2000 Hz. Six muscles on the leg (vastus lateralis, vastus medialis, biceps femoris, gastrocnemius, soleus, and tibialis anterior) and the latissimus dorsi muscle on the back were selected as signal collection points, distinguishing between left and right sides. Figure 3 illustrates the positions where the sensors were attached. Before the experiment began, the selected areas were cleaned with medical alcohol to ensure effective acquisition of the sEMG signals. It should be noted that the sensors must be firmly attached to the muscles to prevent movement of the sEMG signal collector during the collection process. The distance between two sensors must be greater than 30 mm to reduce cross-talk between sensors.

For IMU signal acquisition, STT-IWS2 wireless inertial measurement units (STT Systems, Donostia-San Sebastián, Spain) were used to collect lower limb motion signals at a sampling frequency of 200 Hz. The IMU sensors were attached to the lateral side of the thigh and the anterior side of the shank, respectively. Each sensor was firmly fixed to the lower limb using elastic straps and Velcro to minimize sensor displacement during the experimental trials. For the thigh-mounted IMU, the local *X*-axis was oriented along the femoral direction from the knee joint to the hip joint, whereas for the shank-mounted IMU, the local *X*-axis was oriented along the tibial direction from the ankle joint to the knee joint. The local *Z*-axis of each IMU was positioned as perpendicular to the skin surface as possible. The sensor attachment positions are shown in Figure 3.

After the IMU sensors were attached, a calibration procedure was performed before formal data acquisition. During calibration, each participant was required to maintain a static upright reference posture, and the orientation matrices of the thigh and shank IMUs were recorded using the iSen system (inertial motion analysis software, STT Ingeniería y Sistemas, S.L., Donostia-San Sebastián, Spain).

To ensure temporal consistency of the experimental data, a synchronizer is used to control the computers. The synchronizer allows a single operator to control both computers simultaneously. When the operator instructs one computer to start recording data, the acquisition of sEMG and IMU signals begins simultaneously; when the operator instructs one computer to stop recording data, the acquisition of sEMG and IMU signals ends simultaneously.

In this study, a total of 16 participants were recruited, all of whom had normal leg mobility without any muscle pain or discomfort. Demographics of the participants as shown in Table 1.

All participants were informed and signed a consent form prior to participating in the experiments. Each participant performed three actions—squatting, asymmetrical stance, and single-knee kneeled position—in sets of 10 repetitions, completing three sets for each action. Each participant also performed three actions—crouching advance, walking on flat ground, and running—for 15 s per set, completing three sets for each action. During the crouching advance, an overhead obstacle was placed along the participant’s movement path, with its lower edge 1.6 m above the ground, to simulate a low-ceilinged environment. All participants performed the six movements in the fixed sequence: squatting, asymmetrical stance, single-knee kneeled position, crouching advance, walking on flat ground, and running. After completing each set of actions, participants were given a 3 min rest period to prevent muscle fatigue from interfering with the sEMG signals.

### 2.2. Data Processing

**Figure 4:**
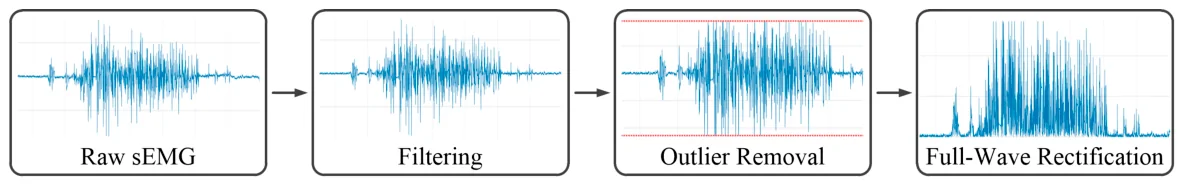
sEMG Preprocessing.

Preprocess the acquired sEMG signals. An 8th-order Butterworth bandpass filter with a frequency range of 10–800 Hz was designed to filter the original signal and eliminate high-frequency noise and unwanted signals. The purpose of outlier detection is to identify and eliminate outliers. The absolute values of all signals within a gait cycle are calculated, and the processed signals are sorted in ascending order. The threshold for identifying outliers is set at the signal value corresponding to the 99th percentile of the sorted data. Any data exceeding this threshold was replaced with the threshold value rather than being deleted. Although this operation may suppress a small number of valid high-amplitude sEMG samples, it can effectively reduce the interference caused by isolated extreme amplitudes resulting from transient noise or electrode disturbance. Full-wave rectification converts the negative half-wave portion of a signal into a positive half-wave portion, making the signal easier to process and analyze. The sEMG preprocessing method used in this study is shown in Figure 4.

During the motion process, the sEMG signals of the lower limbs exhibit good periodicity, with each period containing both resting and active states. However, these complete segments of information typically include a significant amount of noise and irrelevant data. To accurately extract signal features, it is necessary to perform activity segment extraction on the acquired signals. Among the six selected actions, flat ground walking, running, and crouching advance are continuous actions, so the entire duration of these actions is considered as the activity segment. For squatting, asymmetrical stance, and single-knee kneeled position, specific activity segments need to be extracted.

**Figure 5:**
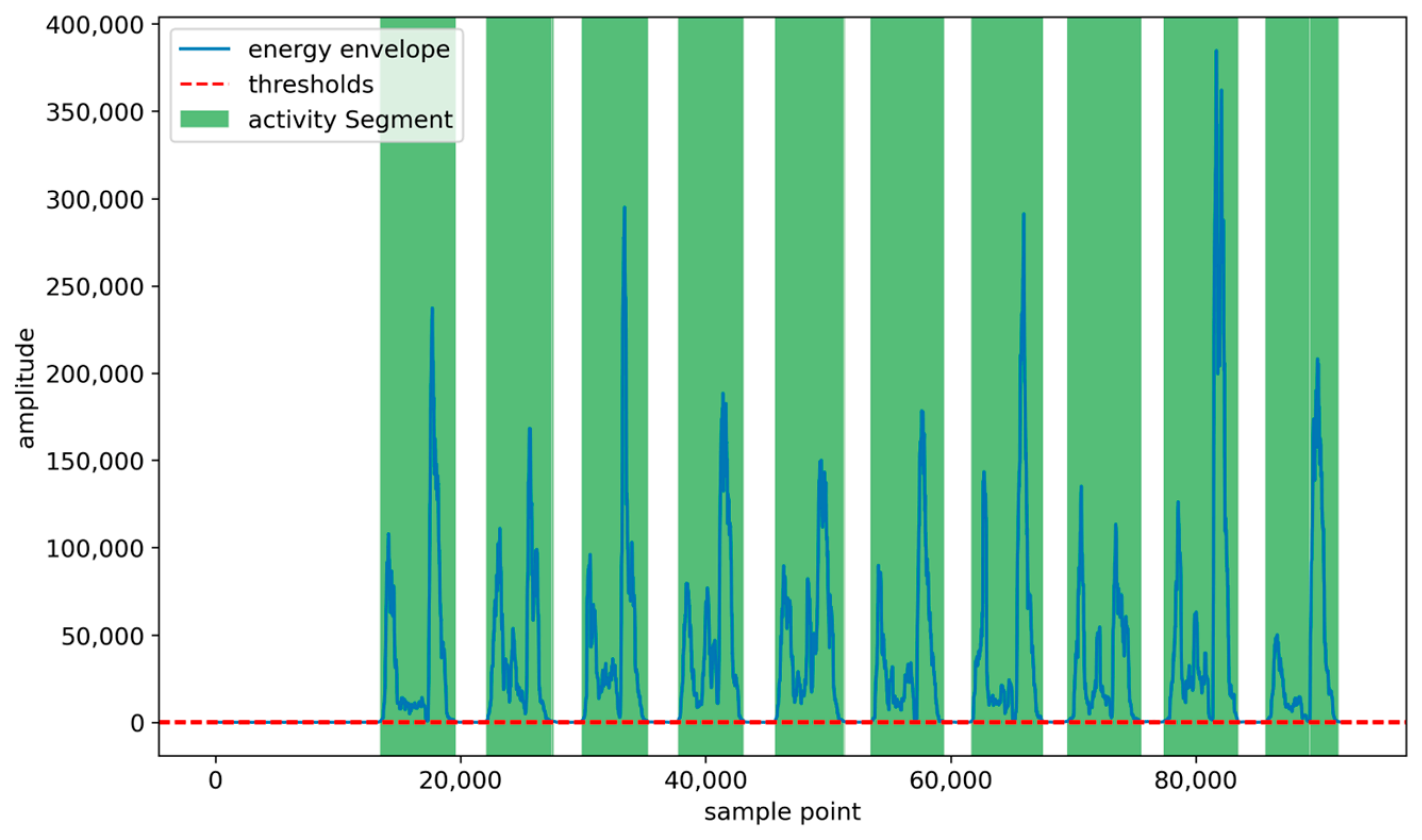
Energy Envelope and Segment Extraction for single-knee kneeled position.

The envelope of the acquired sEMG signals is extracted. Based on the extracted en-velope, the signal amplitude was squared and then smoothed using a moving-average filter with a window length of 300 samples to obtain the sEMG energy envelope, which reflects the temporal variation in signal energy. Then, activity segment extraction is performed on the sEMG and IMU signals using an energy threshold method. The same activity-segment extraction procedure was applied to all participants. For each participant and trial, the mean energy-envelope value during the resting period was calculated, and 1.1 times this value was defined as the activity threshold. Signal portions above the threshold were identified as active states, whereas those below the threshold were classified as resting states. For each movement, the muscle channel exhibiting the most pronounced energy fluctuation was selected as the reference channel, and the detected temporal boundaries were subsequently applied to all synchronized sEMG and IMU channels. For example, Figure 5 shows the energy envelope and activity segment extraction for the single-knee kneeled position action.

After extracting the activity segments from the signals, the sEMG signals are processed using the Empirical Mode Decomposition (EMD) algorithm, which decomposes the raw sEMG signals into a sum of several Intrinsic Mode Functions (IMFs).

In empirical mode decomposition, the sifting algorithm is used to extract the intrinsic mode functions step by step. Construct the upper envelope $U_{k}\left(t\right)$ and the lower envelope $L_{k}\left(t\right)$ based on the local maxima and local minima of the signal to be decomposed, and compute the local mean for the kth sifting $m_{k}\left(t\right)$, as shown in Equation (1).

(1) $$m_{k}\left(t\right)=\frac{\left(U_{k}\left(t\right)+L_{k}\left(t\right)\right)}{2}$$

Subtract the local mean from the current signal time series to obtain a new signal time series.

(2) $$h_{k}\left(t\right)=h_{k-1}\left(t\right)-m_{k}\left(t\right)$$

In Equation (2), $h_{k-1}\left(t\right)$ and $h_{k}\left(t\right)$ represent the signals after the k − 1st and kth sifting, respectively. Repeat the above steps. A common criterion for terminating the sifting process is that, after the algorithm has extracted k − 1 eigenmodes, the resulting residual is also an eigenmode or a monotonic function; at this point, the iteration ends. During this iterative process, multiple eigenmodes can be obtained.

The IMF1 signals extracted using the EMD algorithm are selected, as they retain the richest features of the original signal, and their characteristic values are extracted from the sEMG signals.

The IMU signals extracted in this study are knee joint angles and angular velocities, so there is no need to perform EMD on the IMU signals.

### 2.3. Feature Extraction

The goal of feature extraction is to derive discriminative feature vectors from the complex sEMG signals, enabling the differentiation of various lower limb movement patterns. Features in the time-frequency domain allow for the distinction of different movement patterns. Each movement pattern exhibits distinct sEMG characteristics in the time-frequency domain, which can be utilized to build classification models for recognizing and differentiating various movement patterns.

In this work, six different features are extracted in each step of the sliding window. The features used are root mean square (RMS), waveform length (WL), median frequency (MF), mean absolute value (MAV), mean power frequency (MPF) and sample entropy. These features are commonly used for extracting sEMG signal characteristics. By incorporating all these features in a single feature vector, better results are obtained compared to using only one or a few features [26].

In this experiment, the sEMG signal sampling frequency is 10 times that of the IMU signal. Therefore, when extracting feature values using the sliding window, a window size of 200 and a step size of 10 are set. Since the sEMG and IMU sampling frequencies were 2000 Hz and 200 Hz, respectively, the interval between adjacent sEMG feature vectors was 5 ms, equal to the IMU sampling interval. The timestamp at the end of each sEMG window was used to temporally match the corresponding IMU sample, and unmatched samples at the sequence boundaries were discarded to ensure one-to-one correspondence between the two signal modalities. Before performing sliding window segmentation, the data had already been partitioned into training, validation, and held-out test subsets. The training, validation, and held-out test subsets were then processed separately and divided into windows. The knee joint angle and angular velocity from the IMU signal are extracted and combined with the sEMG signal features to form a fused signal.

### 2.4. Mutual Information Feature Extraction

sEMG signals not only reflect the recruitment intensity of individual muscles, but the interactions between channels also provide deeper insights into the central nervous system’s logic for the coordinated regulation of multiple muscles. While traditional linear correlation coefficients can only capture synchronous firing between channels, MI is a nonlinear metric based on information theory that can quantify the statistical dependencies shared by different muscle groups during specific movements, thereby effectively characterizing neurally driven cooperative properties.

For any two distinct sEMG channel sequences *X* and *Y*, their mutual information *I(X;Y)* can be expressed in terms of entropy as shown in Equation (3),

(3) $$I(X;Y)=\sum_{x\in X}\sum_{y\in Y}p(x,y)\log\frac{p(x,y)}{p(x)p(y)}$$

where p(x,y) denotes the joint probability distribution of the sEMG channel variables *X* and *Y*, while p(x) and p(y) denote the marginal probability distributions of *X* and *Y*, respectively. The variables *x* and *y* represent possible observations of *X* and *Y*.

MI can be understood as the amount of information a random variable contains about another random variable, or as the reduction in uncertainty of one random variable resulting from knowledge of the other [21]. Within the framework of entropy, this formula can be equivalently expressed as:

(4) I(X;Y)=H(X)+H(Y)−H(X,Y)

Here, *H(X)* and *H(Y)* represent the marginal entropies of each channel, respectively, indicating the average uncertainty of the signal; *H(X,Y)* represents the joint entropy. A larger value of *I(X;Y)* indicates that channel *X* contains more information about channel *Y*, meaning that the degree of synergistic coupling between the two muscles during the current movement is higher. Based on Equation (4), we extract the MI features among the muscles on the left side (the six muscles of the left leg and the left latissimus dorsi). A higher mutual information value indicates stronger interdependence or correlation between the muscles.

In this study, mutual information between sEMG channels was numerically estimated using the Kraskov–Stögbauer–Grassberger k-nearest-neighbor estimator for continuous variables; this method does not require predefined bin numbers, bin boundaries, kernel functions, or kernel bandwidths. The estimator used the Chebyshev maximum norm in the joint space, and the number of nearest neighbors was set to k = 5. The random seed was fixed at 42 to ensure reproducibility, negative estimates caused by finite-sample variation were truncated to zero, and no additional analytical bias correction was applied. Mutual information was calculated separately within each 200-sample sliding window.

The primary difference between squats and single-knee kneeled position lies in whether the movements of the left and right legs are synchronized. During single-knee kneeling, the legs are in an asymmetrical position: one leg bends forward at the knee, while the other bends backward, with the knee touching the ground directly. In contrast, during a squat, both legs remain largely symmetrical; both knees bend and push forward simultaneously, with the knees performing only a bending motion without directly touching the ground.

Walking on flat ground is a typical alternating symmetrical movement. Running exhibits the same symmetry as walking, differing only in the rhythm and amplitude of the contact and flight phases. However, due to the forward lean of the body and the extremely low center of gravity during a forward bend, the symmetry of lower limb movements in this motion is significantly weaker than in walking or running.

When extracting MI features, we focus on the mutual information between the symmetrical muscles of the left and right legs, as shown in Equation (5). We extract the mutual information between corresponding left and right muscles and the absolute difference in physical metrics; these features reflect lower limb imbalance during movement. We use the differences in RMS and MPF between the symmetrical muscles of the left and right legs to extract muscle symmetry features (MSF), as shown in Equations (6) and (7).

(5) $${MSF}_{MI}=H(L)+H(R)-H(L,R)$$

(6) $${MSF}_{RMS}=\left|{RMS}_{L}-{RMS}_{R}\right|$$

(7) $${MSF}_{MPF}=\left|{MPF}_{L}-{MPF}_{R}\right|$$

Here, *H(L)* represent the marginal entropies of left leg channel, *H(R)* represent the marginal entropies of right leg channel, *H(L,R)* represents the joint entropy. ${\;RMS}_{L}$ and ${RMS}_{R}$ represents the RMS feature extracted from the left and right leg’s sEMG signal, ${MPF}_{L}$ and ${MPF}_{R}$ represents the MPF feature extracted from the left and right leg’s sEMG signal.

**Figure 6:**
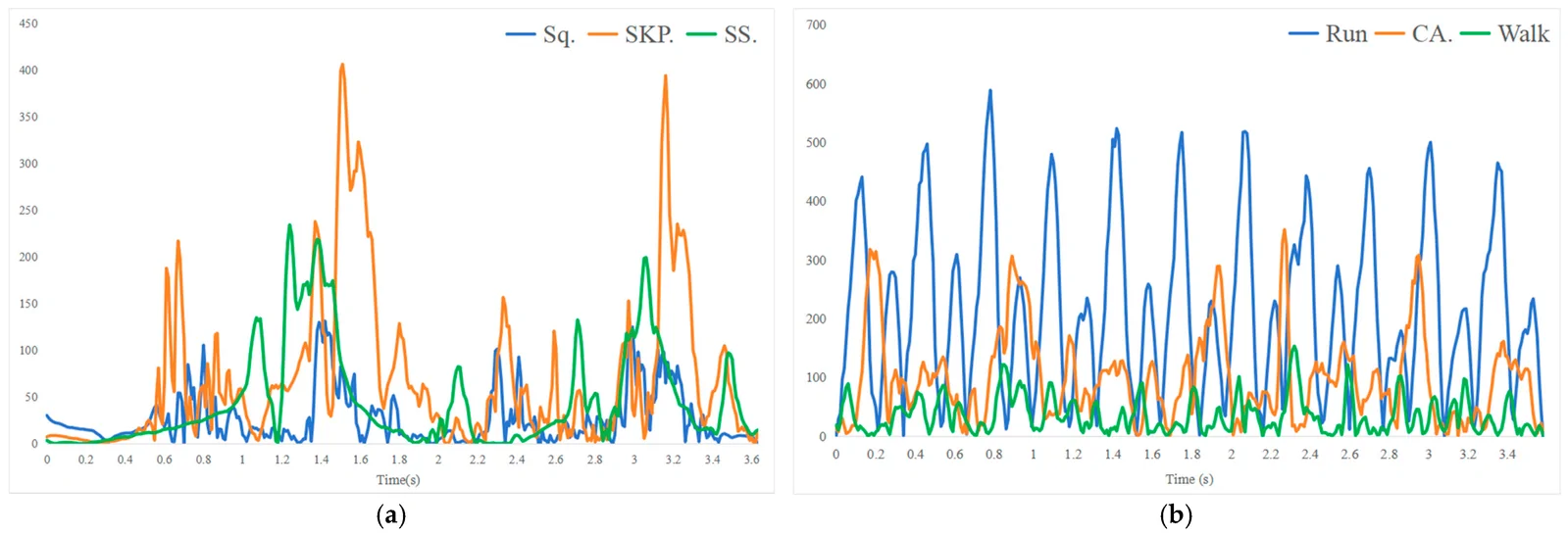
(**a**) Comparison of ${MSF}_{RMS}$ of the Vastus Lateralis During Squat, Single-knee Kneeled and asymmetrical stance Position (**b**) Comparison of ${MSF}_{RMS}$ of the Vastus Lateralis During Run, Stands for crouching advance and Walk Position.

When there is asymmetry between the muscles on both sides during movement, the MSF characteristics change significantly, as shown in Figure 6.

After feature extraction, min–max normalization was independently applied to each feature dimension. For the RMS, WL, MF, MAV, MPF, sample entropy, MI, and MSF features, the minimum and maximum values were estimated exclusively from the training data in each evaluation run. These normalization parameters were then applied un- changed to the corresponding validation and held-out test data.

### 2.5. Dataset Construction

For classification tasks, building an ideal dataset requires filtering out redundant data [34,35]. For signal features extracted from multi-channel sEMG signals under multiple movement patterns, We employ the RWFS + CMI method to fuse and reduce the dimensionality of multi-source signal features.

The RWFS method not only considers information content but also accounts for the importance of the rate of change in determining the information content of features [36]. The weighting is calculated as shown in Equation (8).

(8) $$W(f_{k},f_{j},l_{i})=\sum_{l_{i}\in L}\left[I(f_{k};l_{i})\times \sum_{f_{j}\in S}W(f_{k},f_{j},l_{i})\right]=\sum_{l_{i}\in L}\left\{I(f_{k};l_{i})\times \sum_{f_{j}\in S}\left[\frac{I\left(f_{k};l_{i}|f_{j}\right)}{I(f_{k};l_{i})}+\frac{I\left(f_{j};l_{i}|f_{k}\right)}{I(f_{j};l_{i})}\right]\right\}$$

Here, F denotes the entire feature set, S denotes a subset of selected features, and L denotes the label set. $f_{k}\in F-S,f_{j}\in S,l_{i}\in L$.

CMI is capable of selecting features that are relevant to the target and complementary to the already selected features. CMI helps reduce redundancy and promotes diversity among the selected features, which is particularly beneficial for high-dimensional biomedical datasets [19]. As shown in Equation (9).

(9) $$I\left(X;Y|Z\right)=\sum_{x\in X}\sum_{y\in Y}\sum_{z\in Z}p(x,y,z)\log\frac{p\left(x;y|z\right)}{p\left(x|z\right)p\left(y|z\right)}$$

Here, *X* represents the candidate features, *Y* represents the target variable, and the conditional mutual information between them is conditioned on a set of existing features *Z*. Here, p(x,y,z) is the joint probability distribution of the features *X*, the target *Y*, and the conditioning set Z.

RWFS and CMI were integrated through an iterative feature-selection procedure. At each iteration, the original RWFS criterion was first used to evaluate each candidate feature by considering its relevance to the class-label set, the variation in conditional information, and its redundancy with the currently selected features. Meanwhile, the conditional mutual information between each candidate feature and the class-label set, conditioned on the complete selected feature subset, was calculated to quantify the additional discriminative information provided by that feature. Because the RWFS and CMI scores have different numerical ranges, they were normalized separately and then assigned equal weights to obtain the final fused score of each candidate feature. The feature with the highest fused score was added to the selected subset and removed from the candidate set, after which the RWFS and CMI scores of the remaining candidate features were recalculated based on the updated selected subset. This iterative procedure continued until the cumulative normalized fused weight of the selected features first reached or exceeded 95%.

By combining the two methods, we ensure that the primary features are retained while eliminating a large amount of noise and unimportant components [31,32], and simultaneously assign higher priority to features whose weights fluctuate significantly when symmetry is broken.

In the experiment, sEMG signals from 14 muscles were collected. After EMD of the sEMG signal for each muscle, the first IMF signal was selected as the preprocessed sEMG signal. A total of 84-dimensional basic features were extracted, along with 21-dimensional cross-informational features between muscles on the left side, 7-dimensional symmetrical muscle cross-informational features, and 14-dimensional symmetry difference features. The extracted features were processed using the RWFS + CMI analysis method. All final feature weights obtained were normalized. The features were ranked in descending order according to their normalized weights, and the cumulative sum of the normalized weights of the selected features was calculated, as shown in Equation (10).

(10) $$CR(k)=\frac{\sum_{i=1}^{k}w_{i}}{\sum_{j=1}^{m}w_{j}}\times 100\%$$

**Figure 7:**
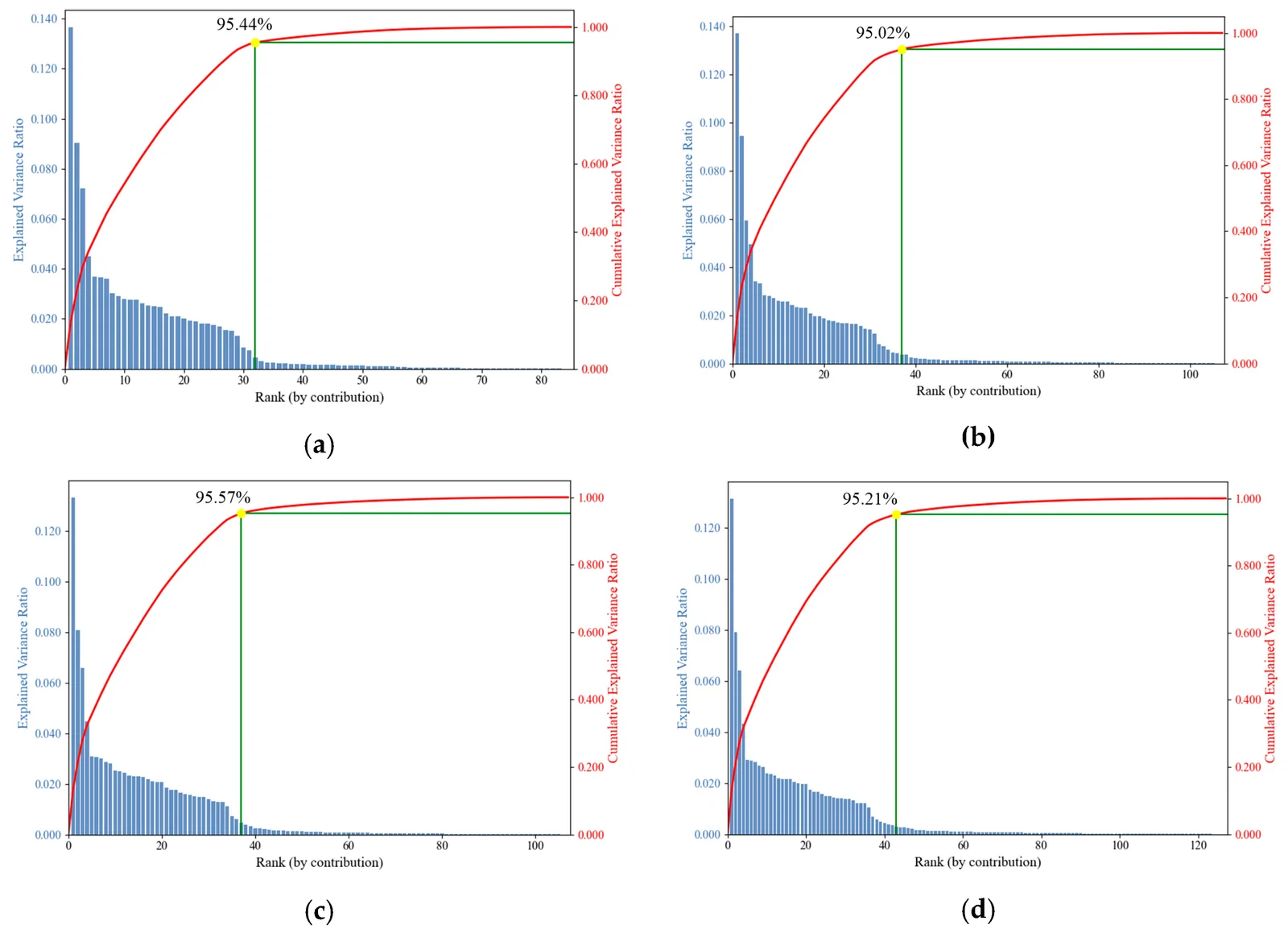
Results of RWFS + CMI Processing (**a**) Dimension reduction results for Fea (**b**) Dimension reduction results for Fea + left leg MI features (**c**) Dimension reduction results for Fea + MSF (**d**) Dimension reduction results for ALL features.

$w_{i}$ represents the final normalized weight of the *i*-th feature obtained using RWFS + CMI, *m* represents the total number of features, and *k* represents the top *k* high-weight features retained, CR(k) stands for the cumulative sum of feature weights. When the cumulative sum first reached or exceeded 95%, the corresponding top *k* features were selected to construct the dimension-reduced feature dataset. The cumulative sum and the normalized weights of each component are shown in Figure 7.

After dimensionality reduction, this study established a total of 8 datasets: the top 32 dimensions of the basic sEMG signal features accounted for 95.44% of the cumulative normalized feature weight, this feature set was denoted as “Fea”; the top 37 dimensions of the basic sEMG signal features combined with left leg MI features accounted for 95.02% of the cumulative normalized feature weight, this feature set was denoted as “Fea + MI”; and the top 38 dimensions of the basic sEMG signal features combined with MSF accounted for 95.57% of the cumulative normalized feature weight, this feature set was denoted as “Fea + MSF”; and the full sEMG signal feature dataset, with the top 43 dimensions, accounted for 95.21% of the cumulative normalized feature weight, this feature set was denoted as “Fea + MI + MSF”. For the four distinct sEMG signal feature datasets, IMU signals were fused to form four sets of fused signal datasets. After merging the signal features, independent labels were assigned to each action.

To avoid information leakage, the RWFS + CMI feature selection procedure was performed only on the training set, and the selected feature indices were then applied to the test set.

## 3. Classification Methods for Lower Limb Movements

The goal of this phase is to use the feature values derived from the extracted sEMG signals and IMU signals to compile a dataset. This dataset will then be divided into training and testing sets. The training set will be used to train a machine learning algorithm, and the testing set will be utilized to validate the classification performance of the algorithm. A CNN-LSTM-Attention machine learning algorithm has been selected for classifying human lower limb movements.

### 3.1. Selection of Classification Algorithm

CNN-LSTM-Attention combines convolutional neural network (CNN), Long Short-Term Memory networks (LSTM), and attention mechanisms. It demonstrates strong potential and advantages in time series classification.

To thoroughly evaluate the performance and effectiveness of the CNN-LSTM-Attention algorithm, this paper compares it with traditional machine learning methods. In addition to comparing CNN algorithms and CNN-LSTM algorithms, three classic machine learning algorithms have been selected: Random Forest (RF), Support vector machine (SVM), and Back Propagation Neural Network (BPNN).

In the CNN-LSTM-Attention algorithm, CNN is used to extract local features from input data, identifying patterns or features within sequences. LSTM handles time series data, capturing long-term dependencies within sequences. The attention mechanism enhances the model’s focus on important features by weighting key information. The weighted sum of LSTM outputs based on attention weights is passed through a fully connected layer for final classification. Therefore, the CNN-LSTM-Attention algorithm effectively handles complex sequence data classification tasks.

In this study, sEMG signals and IMU signals contain spatiotemporal information. Lower limb movements involve various complex patterns, including gait, actions, and posture changes. Using the CNN-LSTM-Attention model allows for a more comprehensive consideration of the multidimensional information in sEMG signals, thus enhancing the accuracy of lower limb movement pattern classification.

### 3.2. Theoretical Background of the Classification Algorithm

**Figure 8:**
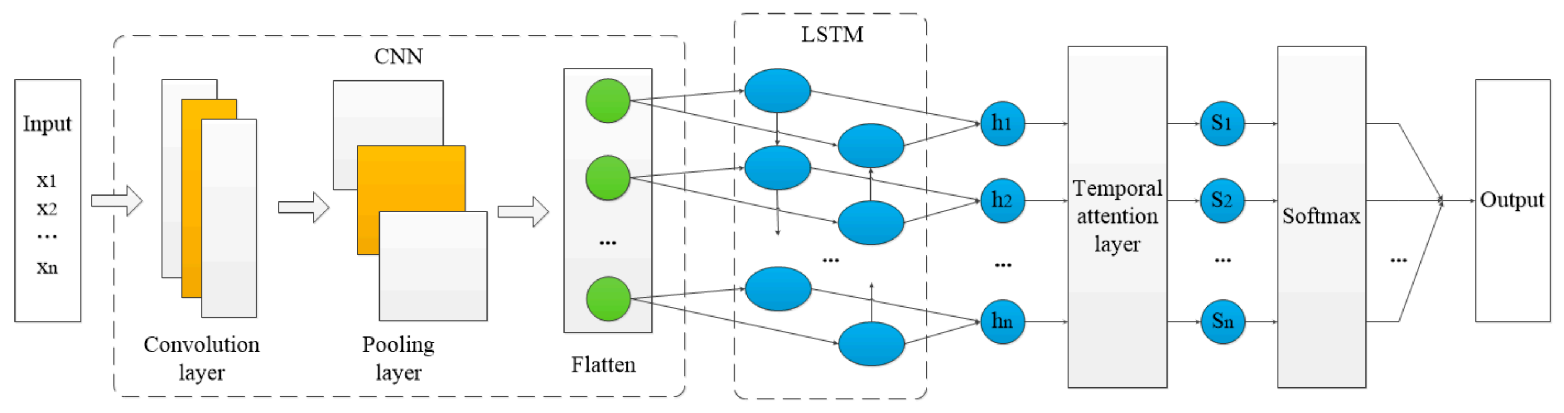
CNN-LSTM-Attention principle.

CNN-LSTM-Attention combines convolutional neural network (CNN), Long Short-Term Memory networks (LSTM), and attention mechanisms, demonstrating strong potential and advantages in time series classification. The workflow of the CNN-LSTM-Attention algorithm is illustrated in Figure 8.

In this study, the input of the model is a dataset composed of feature values extracted from the sEMG signals and IMU signals.

(11) $$X=\{x_{1},x_{2},\cdots ,x_{n}\}$$

Convolutional Neural Networks (CNN) are feedforward neural networks with convolution operations and deep structures. They consist of three types of layers: convolutional layers, grouping layers, and fully connected layers. Convolutional layers and pooling layers generate feature maps. Each plane in one layer is composed of one or more planes from the subsequent layer. In the feature extraction stage, each network layer takes the output of the previous layer as input and passes its own output to the next layer. The nodes in a plane are connected to a small region of nodes in the previous layer. The data set is inputted into the CNN layers. Features are extracted in convolutional layers and pooled in maximum pool layers to reduce dimensionality while retaining significant feature information.

Long Short-Term Memory Networks (LSTM) are a type of recurrent neural network architecture (RNN). LSTMs introduce memory cells that store and pass information. These memory cells allow the network to retain long-term information, which is crucial for capturing dependencies in long-sequence data. LSTMs control the flow of information through gating units, determining which information should be forgotten, added to the memory cell, and read from the memory cell. This unique gating mechanism endows LSTM with powerful learning and expression capabilities. The reduced dimensionality feature data serve as input to the LSTM layer, where the neural network is trained to automatically learn sequence features. Each recurrent neural unit outputs a hidden state H at the end.

(12) $$H=\{h_{1},h_{2},\cdots ,h_{n}\}$$

Through the attention mechanism, each hidden state (H) is assigned an attention score (S), and the attention layer receives the output from the LSTM layer and calculates the attention weights for each time step. These weights represent the contribution of each time step to the final prediction. Finally, the model computes the weighted sum and obtains the final prediction result. The calculation formula is as follows.

(13) $$S=\{s_{1},s_{2},\cdots ,s_{n}\}$$

(14) $$d_{i}=a_{i}h_{im}$$

(15) $$a_{i}=\frac{exp(s_{i})}{\sum_{1}^{n}exp(s_{i})}a_{i}=\frac{exp(s_{i})}{\sum_{1}^{n}exp(s_{i})}$$

(16) $$Q=tan\;h(h_{n}W_{q})$$

(17) $$K_{i}=tan\;h(h_{i}W_{k})$$

(18) $$s_{i}=\frac{K_{i}Q^{T}}{\sqrt{m}}$$

In the formula, $d_{i}$ denotes the context vectors, *Q* is the query vector, $s_{i}$ is the attention scores of hidden units, $a_{i}$ and $K_{i}$ are attention weights and key vectors, *m* denotes the dimension of the query vector *Q*, $W_{k}$ and $W_{q}$ are trainable weight matrices. The attention scores calculated from the LSTM hidden states are normalized using the softmax function to obtain attention weights, with each weight ranging from 0 to 1 and all weights summing to 1. These weights are learned during model training and are used to compute the weighted representation of temporal features.

### 3.3. Data Evaluation Methods

To reduce the influence of participant selection and model randomness, a repeated subject-independent hold-out evaluation was conducted. The experiment was repeated 10 times, and a different participant was selected without replacement as the held-out test subject in each run. All data from the held-out participant, including data from all six movement classes, were excluded from feature selection, model training, validation, and hyperparameter adjustment and were used exclusively for independent testing. Therefore, 10 different participants served as unseen test subjects across the 10 experimental runs.

For the remaining 15 participants in each run, the data were divided into training and validation sets according to the characteristics of the movements. For the three discrete movements, squatting, asymmetrical stance, and single-knee kneeled position, 10 activity segments were extracted from each set, of which 9 were assigned to the training set and the remaining 1 was assigned to the validation set. For the three continuous move- ments, crouching advance, walking, and running, the continuous activity segments were first divided into training and validation subsets at a ratio of 90% to 10%, after which sliding-window generation and feature extraction were performed separately within each subset.

The data-processing order was strictly controlled to prevent information leakage. Participant and trial partitioning was performed before sliding-window generation. The training, validation, and held-out test subsets were processed separately, and sliding win- dows were generated independently within each subset so that no window crossed a sub- set boundary. Feature extraction was then performed separately for each subset. Normal- ization parameters and RWFS+CMI feature-selection parameters were estimated using only the training data. Therefore, no raw samples were shared among the training, vali- dation, and test subsets.

Within each run, the same held-out participant and the same training validation partitions were used for all feature combinations and comparison algorithms. The final evaluation metrics were calculated as the mean and standard deviation across the 10 runs.

Evaluation metrics for pattern classification tasks are used to measure model performance, determining its classification ability and accuracy. To compare the results of lower limb movement pattern classification under different conditions, this study selects confusion matrix, accuracy, precision, recall, and F1 score as evaluation metrics for classification results.

The four elements (TP, TN, FP, FN) in the confusion matrix form the basis for calculating accuracy, precision, recall, and F1 score, As shown in Table 2.

Through the confusion matrix, one can clearly understand the model’s classification performance, including correct and incorrect classifications, thus evaluating the model’s performance in different aspects. These indicators have significant application value in various tasks and problems, helping to measure the effectiveness of classification models. Accuracy, recall, and F1 score are calculated using macro averages. The formulas for accuracy, precision, recall, and F1 score are as follows:

Accuracy refers to the proportion of the number of samples correctly classified by the model to the total sample number:

(19) $$\mathrm{Accuracy}=\frac{TP+TN}{TP+TN+FP+FN}$$

Precision indicates how many of the samples classified as positive categories are true positive categories:

(20) $$\mathrm{Precision}=\frac{TP}{TP+FP}$$

Recall representing how many of the true positive category samples are correctly classified by the model:

(21) $$\mathrm{Recall}=\frac{TP}{TP+FN}$$

F1 score is the harmonic mean of precision and recall, used to comprehensively consider the accuracy and completeness of the classification:

(22) $$F1\;\mathrm{score}\;=2\times \frac{Precision*Recall}{Precision+Recall}$$

## 4. Results

### 4.1. Multi-Source Signal Fusion Results

**Figure 9:**
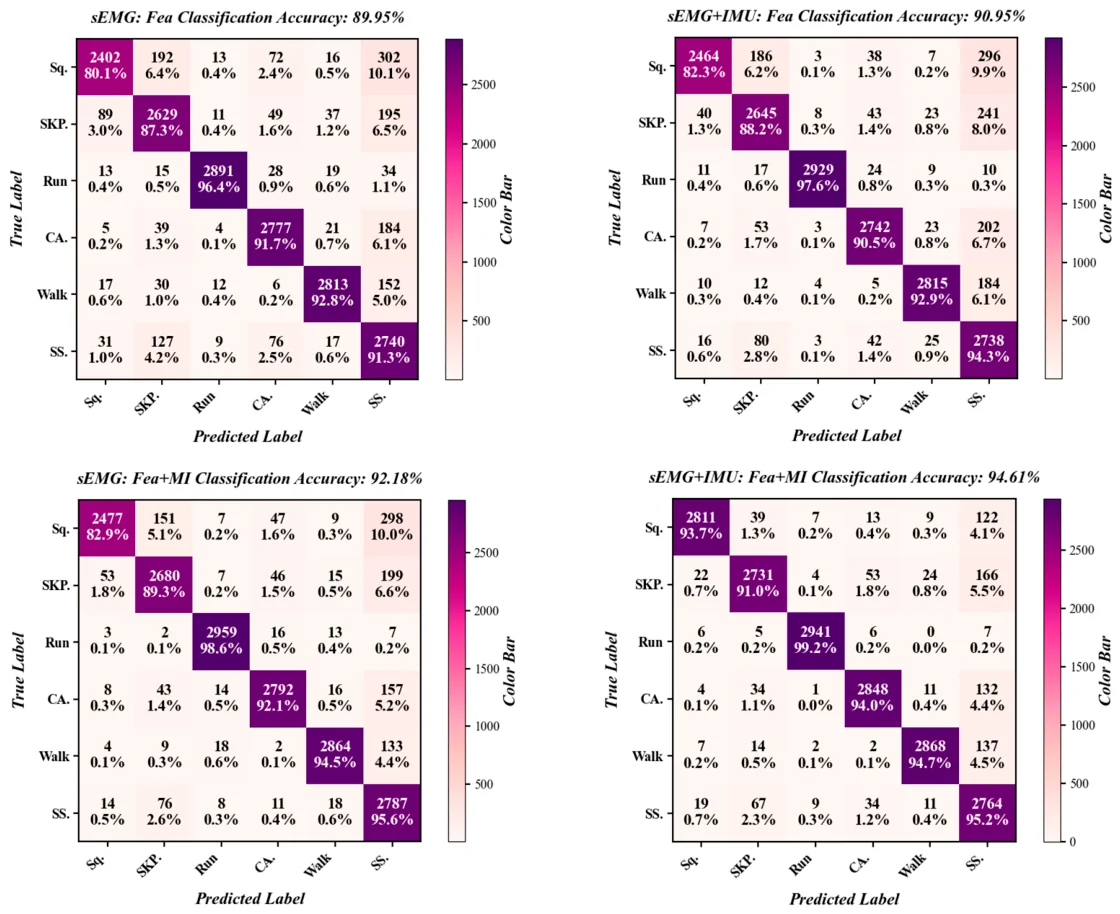

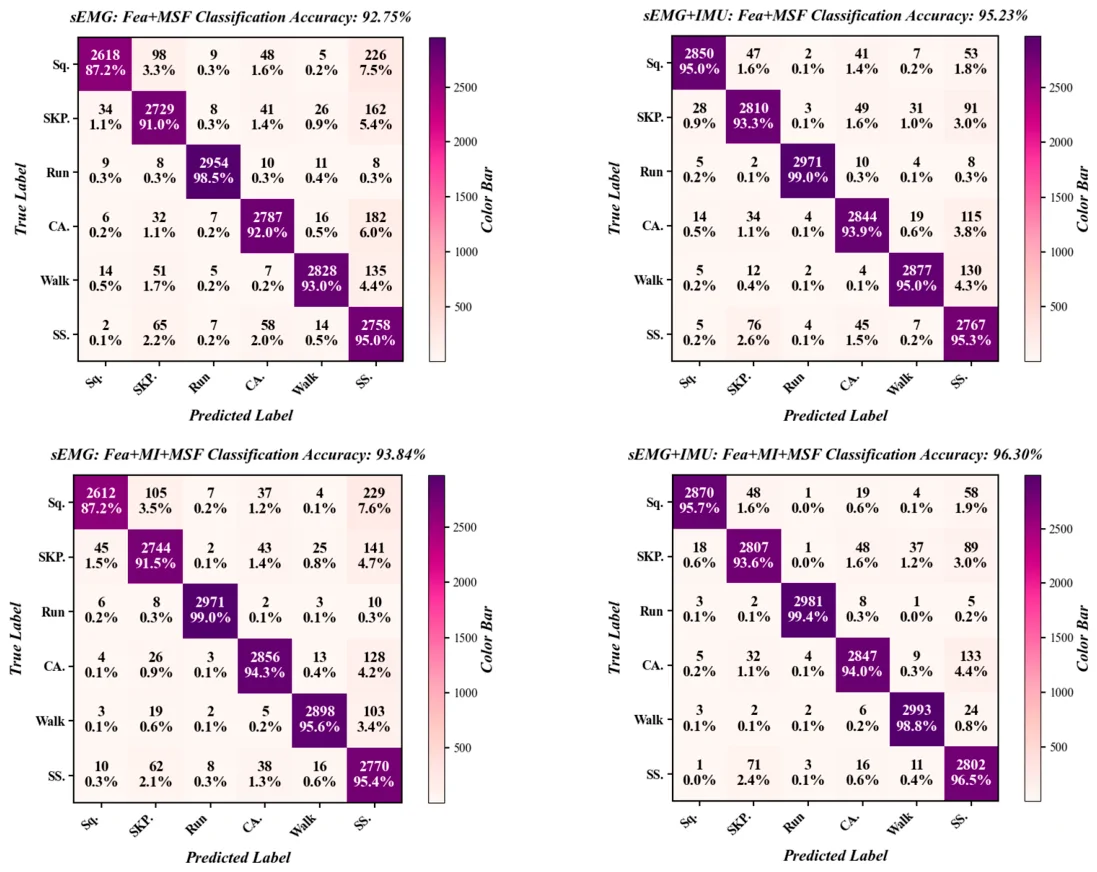
Confusion matrix for different feature combinations.

Confusion matrices were plotted for the classification of sEMG signals and sEMG + IMU signals using different feature combinations, illustrating the classification results on the test set. As shown in the figure, the results for each action can be examined in detail; in the figure, “Sq.” stands for squat, “SKP.” stands for single-knee kneeled position, “CA.” stands for crouching advance, and “SS.” stands for asymmetrical stance. The confusion matrices for different feature combinations are shown in Figure 9.

The proposed method integrates sEMG and IMU information to classify the wearer’s ongoing lower limb movements. The classification accuracy using only sEMG signal features was 89.95%; when MI features were added, the model’s classification accuracy reached 92.18%; when MSF were added, the accuracy reached 92.75%; and when both MI and MSF were added, the accuracy reached 93.84%. The classification performance of the different feature combinations is summarized in Table 3.

For comparison, the RF model used 2000 decision trees, while the SVM adopted an RBF kernel with C = 20 and gamma set to “scale.” The BPNN contained two hidden layers with 512 and 256 neurons and a dropout rate of 0.30. The same data partitions and preprocessing procedures were applied to all models to ensure a consistent comparison.

When sEMG signals were fused with IMU signals, the classification and recognition accuracy using sEMG features + IMU features was 90.95%. Adding MI features increased the model’s classification accuracy to 94.61%; adding MSFs raised it to 95.23%; and incorporating both MI and MSFs simultaneously achieved a classification accuracy of 96.30%.

After incorporating MI features, by capturing the coordination patterns between muscles, the accuracy improved by 0.9~11.4% when classifying relatively complex move- ments such as squats and single-knee kneeling. After incorporating MSF features, which capture differences between symmetrical muscles, the classification accuracy increased by 1.3–2.6% for distinguishing squatting from single-knee kneeling and by 0.2–4.1% for dis- tinguishing crouching advance, walking, and running. Fusion with the IMU signals fur- ther improved the classification accuracy by 1.00–2.48%.

The participant-level classification accuracy was 96.30 ± 0.59%. These results indicate that the proposed method achieved relatively consistent classification performance across the young, healthy participants under the evaluated laboratory conditions.

### 4.2. Analysis of the Classification Results of the Different Algorithms

The CNN-LSTM-Attention model uses a learning rate of 0.001, consists of 3 layers, has 100 hidden units, employs one-dimensional convolutions, and uses max-pooling. These are the optimal parameters used in this study. To verify the classification performance of the CNN-LSTM-Attention model, we compare it with the following other models using the ALL dataset from the sEMG and IMU fused signal group, as shown in Table 4.

From Table 4, the CNN-LSTM-Attention algorithm achieved the highest classification accuracy of 96.30% and also obtained the highest values for recall, precision, and F1 score. Next is the CNN-LSTM algorithm, with an accuracy of 93.29%; the RF algorithm achieved an accuracy of 92.71%; the SVM algorithm achieved an accuracy of 91.88%; the BPNN algorithm achieved an accuracy of 90.51%; and the CNN algorithm achieved an accuracy of 90.45%.

## 5. Discussion

Among the six movements selected in this study, the movement that showed the most significant improvement in classification accuracy after incorporating MI features was the single-knee kneeled position, while the movement with the smallest improvement was walking on flat ground. This is because MI can quantify the statistical dependencies shared by different muscle groups when performing the single-knee kneeled position, which is relatively complex and requires the coordinated action of multiple muscle groups. In contrast, the walking movement is simpler than the others, and the coordination between muscles is less complex, resulting in a more limited improvement.

The inclusion of MSFs enables better differentiation between the “squat” and “single-knee kneeled position” movements. In these two movements, the left leg performs the same action, but the right leg performs completely different actions. The MSFs can accurately reflect the differences between these two movements, thereby improving classification accuracy.

The use of multi-source signal fusion yields better results than relying solely on sEMG signals. This is because multi-source signal fusion leverages the strengths of various signals while avoiding the limitations of a single signal in expressing features.

The classification accuracy with MI features is slightly lower than that with MSFs. A possible reason is that MSFs provide greater discriminative power across the six movements selected in this study. When using the RWFS + CMI analysis method for data dimensionality reduction, the sEMG feature dataset was reduced from 84 dimensions to 32 dimensions. With the addition of MI features, the dataset was reduced from 105 dimensions to 37 dimensions, resulting in an increase of 5 dimensions, with MI features accounting for 5 of those dimensions. With the addition of MSFs, the dataset was reduced from 105 dimensions to 38 dimensions, resulting in an increase of 6 dimensions, with MSFs accounting for 7 of those dimensions. When both MI and MSFs were incorporated, the dataset was reduced from 126 dimensions to 43 dimensions, resulting in an increase of 11 dimensions, with MI features accounting for 5 dimensions and MSFs accounting for 7 dimensions. It can therefore be inferred that MSFs contribute more significantly to the six motion classification tasks in this study.

One limitation of the current experimental design is that all participants performed these six movements in the same fixed order, which means that movement labels may be associated with the recording order, fatigue, electrode drift, sensor displacement, or baseline shifts over time. Future experiments will randomize or balance the movement sequences across participants.

In future research, we need to collect more data for additional movements, such as obstacle crossing and climbing, which are essential in battlefield scenarios. Additionally, shooting posture is closely related to upper limb motion signals. However, in this study, only back muscle sEMG signals were collected. Collecting sEMG signals from more upper limb muscles could help more accurately differentiate between asymmetrical standing and single-knee kneeled position.

## 6. Conclusions

This study primarily investigated sEMG and IMU signals associated with three specific lower limb movements, with the aim of improving the use of exoskeletons in confined and other specialized environments. This paper employs the CNN-LSTM-Attention deep learning algorithm to distinguish six types of lower limb movements. During the feature extraction phase, this study extracted MI and MSFs to improve the classification accuracy for asymmetric movements. In the dataset construction phase, the RWFS + CMI method was used to perform dimensionality reduction on the feature-based dataset, which not only preserves key features but also assigns higher priority to features with significant fluctuations, resulting in a significant reduction in the dimensionality of multi-source signal features. Based on the comparison of classification accuracy and F1-score, the CNN-LSTM-Attention model achieved a lower limb movement classification accuracy of 96.30% among the 16 young, healthy participants under the evaluated laboratory conditions. Compared to other algorithms, the CNN-LSTM-Attention algorithm achieved the optimal results. Future work will focus on randomized movement sequences and more comprehensive subject-independent validation, such as complete leave-one- subject-out cross-validation across all participants. Meanwhile, more lower limb motion data will be collected to enable more diverse and accurate motion classification. Another important direction will be to integrate the proposed movement-classification method with exoskeleton control and evaluate its performance in practical human–exoskeleton interaction scenarios. However, the current 14-channel sEMG sensor configuration may be difficult to deploy in practical applications because of its relatively high sensor-placement and setup requirements. Therefore, future studies will investigate reduced sensor configurations and identify a smaller set of informative muscle channels while maintaining acceptable classification performance, thereby providing more reliable support for applying lower limb motion classification technology to exoskeleton systems.

## Figures and Tables

**Table 1:** Demographics of the participants.

	Age	Weight (Kg)	Height (cm)	Gender
Participant 1	25	85	186	male
Participant 2	24	70	173	male
Participant 3	24	72	176	male
Participant 4	22	65	168	male
Participant 5	24	77	180	male
Participant 6	23	72	176	male
Participant 7	26	80	185	male
Participant 8	25	65	169	male
Participant 9	25	65	183	male
Participant 10	23	75	185	male
Participant 11	24	59	173	male
Participant 12	24	70	174	male
Participant 13	25	59	171	female
Participant 14	24	61	167	female
Participant 15	25	65	173	female
Participant 16	22	64	170	female

**Table 2:** Confusion matrix.

	Positive	Negative
True	True Positive (TP)	True Negative (TN)
False	False Positive (FP)	False Negative (FN)

**Table 3:** Classification accuracy of the multi-source signal fusion model using a CNN-LSTM-Attention model.

	Accuracy	Precision	Recall	F1 Score
sEMG: fea	89.95 ± 0.81%	0.899 ± 0.008	0.898 ± 0.008	0.898 ± 0.008
sEMG: fea + MI	92.18 ± 0.65%	0.921 ± 0.007	0.921 ± 0.007	0.919 ± 0.007
sEMG: fea + MSF	92.75 ± 0.56%	0.926 ± 0.006	0.927 ± 0.006	0.921 ± 0.006
sEMG: fea + MI + MSF	93.84 ± 0.69%	0.929 ± 0.007	0.932 ± 0.007	0.926 ± 0.007
sEMG + IMU: fea	90.95 ± 0.75%	0.909 ± 0.008	0.910 ± 0.008	0.904 ± 0.008
sEMG + IMU: fea + MI	94.61 ± 0.50%	0.944 ± 0.005	0.946 ± 0.005	0.944 ± 0.005
sEMG + IMU: fea + MSF	95.23 ± 0.53%	0.943 ± 0.005	0.951 ± 0.005	0.946 ± 0.005
sEMG + IMU: fea + MI + MSF	96.30 ± 0.59%	0.960 ± 0.006	0.963 ± 0.006	0.961 ± 0.006

**Table 4:** Accuracy rates of different algorithm classifications.

	Accuracy	Precision	Recall	F1 Score
RF	92.71% ± 0.51%	0.931 ± 0.005	0.970 ± 0.005	0.928 ± 0.005
SVM	91.88% ± 0.49%	0.922 ± 0.005	0.918 ± 0.005	0.920 ± 0.005
BPNN	90.51% ± 0.42%	0.909 ± 0.004	0.905 ± 0.004	0.906 ± 0.004
CNN	90.45% ± 0.48%	0.919 ± 0.005	0.893 ± 0.005	0.887 ± 0.005
CNN-LSTM	93.29% ± 0.67%	0.935 ± 0.007	0.933 ± 0.007	0.933 ± 0.007
CNN-LSTM-Attention	96.30% ± 0.59%	0.960 ± 0.006	0.963 ± 0.006	0.961 ± 0.006

## Data Availability

The raw data supporting the conclusions of this article will be made available by the authors on request.
